# High EphA2 protein expression in renal cell carcinoma is associated with a poor disease outcome

**DOI:** 10.3892/ol.2014.2196

**Published:** 2014-05-28

**Authors:** JINSHENG XU, JUNXIA ZHANG, LIWEN CUI, HUIRAN ZHANG, SHENGLEI ZHANG, YALING BAI

**Affiliations:** Department of Nephrology, The Fourth Hospital of Hebei Medical University, Shijiazhuang, Hebei 050011, P.R. China

**Keywords:** renal cell carcinoma, receptor tyrosine kinases, EphA2, outcome

## Abstract

The receptor tyrosine kinase, ephrin type-A receptor 2 (EphA2), is normally expressed at sites of cell-to-cell contact in adult epithelial tissues, however, recent studies have shown that it is also overexpressed in various types of epithelial carcinomas, with the greatest level of EphA2 expression observed in metastatic lesions. In the present study, the association between the expression of EphA2 and the outcome of RCC patients was assessed. The high expression level of EphA2 was identified by log-rank test for a statistically significant prediction of the RCC outcome. In an overall multivariate analysis, the high expression level of EphA2 was identified as an independent predictor of RCC outcome. The length of survival of the patients with high EphA2 expression was shorter than that of the patients with a low level of expression (relative risk, 2.304; 95% CI, 1.102–4.818; P=0.027). The analysis of the expression levels of EphA2 in tumor tissues may aid in the identification of the patient subgroup that are at a high risk of a poor disease outcome.

## Introduction

Renal cell carcinoma (RCC) accounts for 2–3% of all cases of malignancy in adults worldwide. In total, >270,000 new cases (2.1% new cases of all cancers) and 100,000 mortalities occur each year (1), and the incidence is increasing (2). RCC is the second most common malignancy in the genitourinary system in China, representing an annual average of 6% of all cases over the last 20 years (3). As RCC is a disease that is associated with few early signs or symptoms, and is commonly diagnosed at an advanced stage (4), the development of novel approaches for outcome prediction is vital in patients with RCC. Certain molecular factors, including vascular endothelial growth factor, interleukin 8 and platelet-derived growth factor, show predictive power for the outcome of RCC (5).

Receptor tyrosine kinases have significant roles in human tumor generation and progression (6). The ephrin (Eph) receptors are the largest receptor tyrosine kinase subfamily and are active in the regulation of cell growth, migration, angiogenesis and survival (7–9). The Eph receptors form two subfamilies, EphA and EphB, with nine EphA receptors (A1–A9) and six EphB receptors (B1–B6), which preferentially bind to the ligands of EphA and EphB, respectively (9). While Eph type-A receptor 2 (EphA2) was initially isolated from a HeLa cell cDNA library (10), EphA1, its main ligand, was originally located in human umbilical vein endothelial cells in a tumor necrosis factor α model (11,12). High expression levels of EphA2 are often found in cancer and have been analyzed in tumor cells using a wide range of cell models and clinical specimens, including those of prostate (13), breast (14) and non-small cell lung (15) cancer. It has been found that EphA2 is highly expressed in several types of human malignant tumor cells and in blood vessels (16), indicating that EphA2 plays a role in tumor development. However, EphA2 does not appear to function simply as a marker, but also as an active participant in malignant progression. To the best of our knowledge, there have been no previous studies on the association between EphA2 and the outcome of RCC.

The aim of the present study was to examine the expression of EphA2 in a series of 62 RCC samples and to assess any association with the disease outcome.

## Materials and methods

### Patients and specimens

In total, 62 patients with RCC were included in this study. The patients were treated in the Department of Urinary Surgery at the Fourth Hospital of Hebei University (Hebei, China) between 2002 and 2007. Of the 62 patients, 36 were male and 26 were female, with an a range of 30–78 years (mean age, 56.18 years). All patients underwent potentially curative surgery without pre-operative therapy. The tumor stages were classified according to the seventh edition of the tumor-node-metastasis (TNM) classification of malignant tumors (17). All patients were followed until mortality or until five years post-diagnosis. A total of 55 patients (88.7%) succumbed during the follow-up. Surgically-removed specimens were routinely fixed in buffered formalin and embedded in paraffin blocks for clinical diagnosis and reclassification for this study. A total of 22 tumor and paired normal tissues were randomly collected from patients during surgery between June 2007 and June 2008, all of which were validated by pathologists. All specimens were snap frozen immediately and stored in liquid nitrogen for RNA and flow cytometry. The study was approved by the Human Tissue Research Committee of the Fourth Hospital of Hebei Medical University. All patients provided written informed consent for the collection of samples and the subsequent analysis.

### EphA2 protein expression analysis by immunohistochemical staining

EphA2 expression was determined by immunostaining using the avidin-biotin complex immunoperoxidase method, which was performed on parallel histopathological sections from the paraffin-embedded tumor section. Subsequent to the blocking of endogenous peroxidase and non-specific reactions, the primary antibody against EphA2 (1:400 dilution; rabbit anti-human polyclonal antibody, sc-924; Santa Cruz Biotechnology, San Diego, CA, USA) was applied to the sections, which were then incubated with biotinylated secondary antibody and DAB reagent; 0.5% 3,3′-diaminobenzidine (Sigma, St Louis, MO, USA) was used as the chromogen. For a negative control, the primary antibody was replaced with mouse immunoglobulin G. Sections of human breast carcinomas known to express EphA2 were included in each staining series as positive controls.

### Evaluation of staining for EphA2

**Figure 1:**
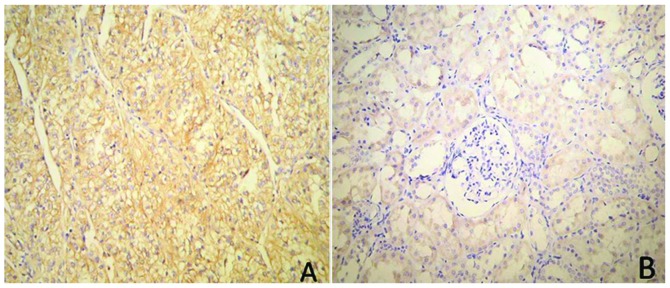
Ephrin type-A receptor 2 (EphA2) immunostaining in renal cell carcinoma (RCC) tissues with (A) high and (B) low expression levels. Cells with a brown-stained nucleus are regarded as positive. Original magnification, ×200.

The stained slides were scored by two investigators who were blinded to the clinical results. The immunostaining results for all receptors were semi-quantified using the H-score (18,19). Briefly, the score was calculated based on the estimates of the percentage of positively-stained RCC cells in each of five intensity categories (0, 1+, 2+, 3+ and 4+). The H-score represents the sum of each of the percentages multiplied by the weighted intensity of staining as follows: H-score = π(i + 1), where i equals 1, 2, 3 and 4 and π varies between 0 and 100%. A score of >100% was defined as high expression and ≤100% as low expression (Fig. 1).

### Total RNA isolation and reverse transcription polymerase chain reaction (RT-PCR)

The total RNA of the tissue specimens was isolated by a SimplyP Total RNA Extraction kit (Bioer Technology Co. Ltd, Hangzhou, Zhejiang, China). The total RNA (1 μg) was reverse transcribed by High-Capacity cDNA Reverse Transcription kits (Applied Biosystems, Foster City, CA, USA) according to manufacturer’s instructions. The primers were synthesized by Sangon Biotech (Shanghai) Co., Ltd. (Shanghai, China). The primers for EphA2 and GAPDH (internal control) were designed as follows: EphA2 forward, 5′-CCAAGTTCGCTGACATCGT-3′ and reverse, 5′-GCCATGAAGTGCTCCGTAT-3′; and GAPDH forward, 5′-ACCACAGTCCATGCCATCAC-3′ and reverse, 5′-TCC ACCACCCTGTTGCTGTA-3′. The RT product (1 μl) was amplified by PCR using the following conditions: One incubation of 5 min at 95°C for PCR; then 28 cycles for the EphA2 primers and 25 cycles for GAPDH at 95°C for 30 sec, 58°C for 45 sec and 72°C for 60 sec, and a final extension at 72°C for 7 min. The PCR product (8 μl) was then electrophoresed on 1.5% agarose gel, and the intensity of the bands was quantified by FluorChem FC2 (Alpha Innotech, San Leandro, CA, USA). EphA2 mRNA expression was determined as a relative intensity of the PCR product bands from the target sequences relative to the intensity of the GAPDH gene. PCR experiments were repeated three times.

### Statistical analysis

Continuous variables were expressed as the mean ± standard deviation. Student’s two-tailed t-test was used to compare EphA2 expression between tumor and normal tissues. The protein expression and clinicopathological parameters were compared by the χ^2^ test. Survival curves were calculated using the Kaplan-Meier method, and comparisons between the curves were made using the log-rank test. Multivariate survival analysis was performed using a Cox proportional hazards model. All statistical analyses were performed using the SPSS 17.0 software (SPSS, Inc., Chicago, IL, USA). For all the statistical tests, P<0.05 was considered to indicate a statistically significant difference.

## Results

### Immunohistochemistry results and correlations between EphA2 expression levels and clinicopathological variables

A total of 62 archival RCC tumor samples with intact clinicopathological materials were initially tested for EphA2 protein expression by immunohistochemistry and correlated with clinicopathological parameters. Among all the samples analyzed, 42 cases (67.7%) demonstrated a high expression level of EphA2 protein, while 20 (32.3%) cases exhibited a low expression level. Positive EphA2 immunostaining was predominantly diffusely distributed throughout the cytoplasm of the RCC tumor cells (Fig. 1). In addition, the χ^2^ test was applied to assess the association between the expression level of EphA2 protein and various clinicopathological variables (Table I). Notably, a high expression level of EphA2 protein was significantly associated with the RCC TNM classification (P=0.001), the size of tumor (P=0.001) and the presence of lymph node metastasis (P=0.022), respectively. However, no significant association was found between the expression level of EphA2 protein and the variables of age and gender (P>0.05).

### EphA2 mRNA and protein expression in RCC tissues

**Figure 2:**
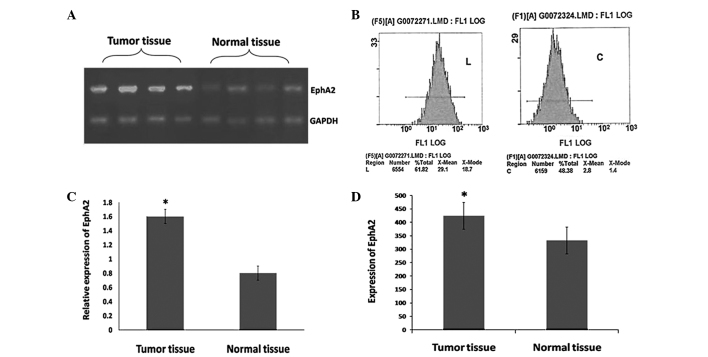
Expression of EphA2 mRNA and protein in 22 paired RCC and normal tissues. The abundance of EphA2 mRNA is shown relative to the levels of GAPDH. The abundance of EphA2 protein was detected by flow cytometry. (A) Reverse transcription polymerase chain reaction (RT-PCR) results for all the samples. (B) Flow cytometry results. (C) Relative expression levels of EphA2 mRNA in the tissue specimens. (D) The protein expression levels of EphA2 in the tissue specimens. Student’s t test demonstrated that the expression levels of EphA2 mRNA and protein in the RCC tissues were significantly higher than those in the normal tissues (both P<0.001). RCC, renal cell carcinoma; EphA2, ephrin type-A receptor 2. ^*^P<0.05 vs. normal tissue

Due to the lack of corresponding normal tissues as a control and with the aim of obtaining the EphA2 mRNA expression pattern, 22 paired freshly collected clinical RCC tissue specimens were further investigated by RT-PCR and flow cytometry. RT-PCR analysis using the EphA2-specific primers indicated that EphA2 mRNA was readily detectable in all the RCC and paired normal tissues. However, the levels of EphA2 mRNA expression were significantly elevated in the RCC specimens with respect to the corresponding normal samples (1.60±0.80 vs. 0.58±0.19, respectively; t=−5.719; P<0.001; Fig. 2A and C). Consistent with the mRNA data, the level of EphA2 protein expression was significantly higher than that in the corresponding normal tissues (424.38±43.14 vs. 332.44±42.83, respectively; t=−5.953; P<0.001; Fig. 2B and D).

### Correlations between clinicopathological variables and the outcome of RCC

All 62 patients were reviewed every six months over a five-year period. None of the patients were lost to follow-up in these five years, and none received adjuvant chemotherapy or radiation therapy following RCC resection. The association between the data collected during the 5-year follow-up and the clinical characteristics was analyzed using the Kaplan-Meier method and the log-rank test. Gender and age were not statistically significant predictors of post-operative survival time, however, tumor size, TNM classification and presence of lymph node metastasis were correlated with survival time in these patients (Table II). As expected, the patients at the various tumor stages had different 5-year survival rates; rates of 23.0% for stage I and 0% for stages II–III were observed. The variations in survival time between these stages were determined to be significantly different (P<0.05) using the log-rank test. Tumor size showed an association with survival rate when patients with a tumor diameter of ≥5 cm were compared to those with a tumor diameter of <5 cm. Additionally, Lymph node metastasis was associated with decreased survival time when patients with lymph node metastases were compared to patients negative for lymph node metastases. These data demonstrate that tumor size, TNM classification and lymph node metastases are good predictors of RCC outcome.

### EphA2 expression and their correlations with RCC survival

**Figure 3:**
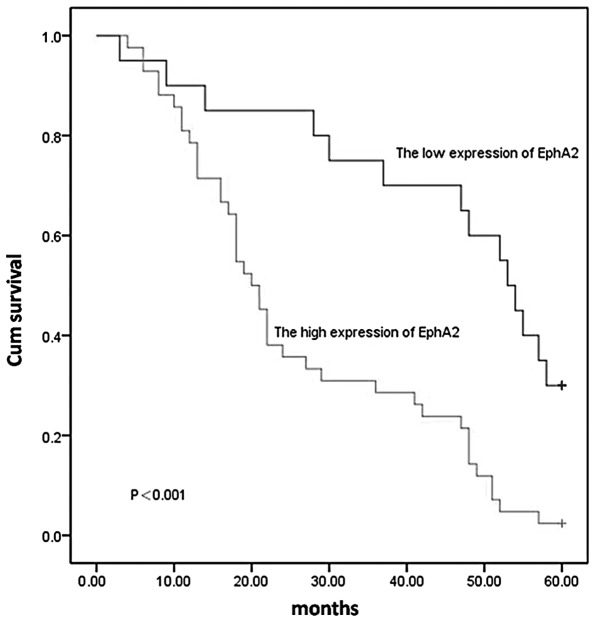
Survival curve according to the protein expression level of ephrin type-A receptor 2 (EphA2) in the renal cell carcinoma (RCC) tissues.

All 62 cases were analyzed for EphA2 staining, and the H-score was calculated. A high expression level of EphA2 in the cancer cells was observed in 42 tumor samples (67.7%). The overall survival rates of the RCC patients with low or high EphA2 levels were compared with the Kaplan-Meier method. A dramatic difference in survival rate was found between the differing expression levels of EphA2 (Table II; Fig. 3). Multivariate analysis was applied using the Cox proportional hazards model with the prediction factors of tumor size, TNM classification, lymph node metastasis and the expression of EphA2. As shown in Table III, the expression of EphA2 was again found to be an independent predictor for RCC outcome. The length of the survival time of the patients with high levels of EphA2 expression was significantly lower than that of the patients with low levels of EphA2 expression (relative risk, 2.304; 95% CI, 1.102–4.818; P=0.027). These data demonstrated the strong predictive power of EphA2 levels on the outcome for patients with RCC.

## Discussion

The level of EphA2 expression in resected RCC tumors was examined in the present study in order to determine its predictive power relative to the outcome of RCC. It was shown that the different levels of EphA2 expression modified RCC survival time in a recessive manner. The patients with RCC lesions exhibiting the high protein expression levels of EphA2 appeared more likely to survive for shorter periods of time. Consistent with a previous study in lung carcinoma (15), the present results support EphA2 expression as an important index of disease progression. As EphA2 can be highly expressed in a broad range of cancer types (20–22), the prognostic utility of this marker may prove significant in the monitoring and clinical management of a large cohort of cancer patients.

Univariate and multivariate analyses indicate that the high expression level of EphA2 in tumor tissues is a prognostic factor in patients with RCC. However, the underlying mechanisms behind the high EphA2 expression level remain unclear. Certain studies have hypothesized that the high EphA2 expression level in tumor cells is due to increased protein stability (23–25). Unstable cell-to-cell contacts could functionally decrease the possibility of EphA2 binding with its membrane-anchored ligands, thus further decreasing ligand-mediated degradation, which contributes to the high expression level of EphA2 in tumor cells. The high level of EphA2 expression was identified to be associated with cancer outcome (22,26).

As a receptor tyrosine kinase, EphA2 at high expression levels not only affects cell proliferation, but also changes the receptors invasive behavior, i.e., the receptors are mislocalized in malignant cells with high expression levels of EphA2, as they are not able to bind their eph ligands, and are therefore not phosphorylated, resulting in increased extracellular matrix adhesions and higher metastatic potential (27). A previous study suggested that the high expression of EphA2 promoted adherent junction destabilization through a signaling pathway for the recruitment of Src kinase, activating LMW-PTP and RhoA GTPase and inhibiting p190RhoGAP (28). EphA2 physically and functionally interacts with ErbB2 to amplify Ras/mitogen-activated protein kinase (MAPK) and RhoA signaling in tumor cells, indicating that EphA2 cooperates with ErbB2/Neu to promote mammary adenocarcinoma tumorigenesis and metastatic progression (29). The association between the high expression level of EphA2 and the RCC survival rates requires further study.

In conclusion, the present study found that a high expression level of EphA2 was found to be an independent prognostic marker for RCC outcome. The analysis of EphA2 expression in tumor tissues may aid in the identification of the patient subgroup that are at a high risk of a poor disease outcome, thereby assisting in refining therapeutic decisions for RCC patients.

## Figures and Tables

**Table I:** Correlations between EphA2 expression and clinicopathological variables in patients with RCC.

		EphA2 expression, n			
				
Characteristics	No. of cases	Low	High	χ^2^	P-value
Age, years				3.302	0.069
<55	24	11	13		
≥55	38	9	29		
Gender				2.070	0.150
Male	36	9	27		
Female	26	11	15		
TNM classification					
I	30	16	14	11.814	0.001
II+III	32	4	28		
Size of tumor, cm (diameter)				10.806	0.001
<5	25	14	11		
≥5	37	6	31		
LN metastasis				5.220	0.022
Negative (N0)	48	19	29		
Positive (N1/2/3)	14	1	13		

RCC, renal cell carcinoma; EphA2, ephrin type-A receptor 2; LN, lymph node.

**Table II:** Univariate analysis of clinical characteristics associated with post-surgical survival in patients with RCC.

Characteristics	No. of cases	5-year survival rate, %	χ^2^	P-value
Age, years			0.004	0.947
<55	24	12.5		
≥55	38	10.5		
Gender			1.298	0.255
Male	36	13.9		
Female	26	7.7		
TNM classification				
I	30	23.0	6.725	0.010
II+III	32	0.0		
Size of tumor, cm (diameter)			8.206	0.004
<5	25	24.0		
≥5	37	2.7		
LN metastasis			8.383	0.004
Negative (N0)	48	14.6		
Positive (N1/2/3)	14	0.0		
Expression of EphA2 protein			16.411	<0.001
Low (negative/weak)	20	30.0		
High (moderate/strong)	42	2.4		

RCC, renal cell carcinoma; EphA2, ephrin type-A receptor 2; LN, lymph node.

**Table III:** Multivariate analysis of prognostic factors associated with post-operational survival in RCC patients with Cox proportional hazards model.

Factors	Relative risk	95% CI	P-value
TNM classification	1.451	0.654–3.222	0.360
LN metastasis	0.830	0.403–1.707	0.612
Size of the tumor	0.933	0.407–2.138	0.870
EphA2	2.304	1.102–4.818	0.027

RCC, renal cell carcinoma; EphA2, ephrin type-A receptor 2; LN, lymph node; TNM, tumor-node-metastasis; CI, confidence interval.
